# Integrating 3D Osteocyte Culture, Microgravity Simulation, and Fluid Flow Reveals Mechanisms of Osteocyte Mechanosensation and Calcium Signaling Altered by Disuse

**DOI:** 10.3390/biom15111534

**Published:** 2025-10-31

**Authors:** Kanglun Yu, Anik Tuladhar, Samuel Dankberg, Caihong Dai, Meghan E. McGee-Lawrence

**Affiliations:** Department of Cellular Biology and Anatomy, Medical College of Georgia, Augusta University, 1120 15th Street, CB1101, Augusta, GA 30912, USA

**Keywords:** osteocyte, mechanotransduction, microgravity, unloading

## Abstract

Osteocytes translate fluid shear stress into biochemical signals critical for bone homeostasis. Here, we combined 3-dimensional (3D) osteocyte culture, microgravity simulation, fluid shear mimicking reloading after disuse, and real-time calcium signaling analysis to elucidate responses of osteocytes under different mechanical environments. Ocy454 cells were seeded onto 3D scaffolds and cultured under static (control) or simulated microgravity (disuse) conditions using a rotating wall vessel bioreactor. Elevated expression levels of *Sost*, *Tnfsf11* (Rankl), and *Dkk1* were detected following disuse, confirming efficacy of the microgravity model. Cell membrane integrity under mechanical challenge was evaluated by subjecting scaffold cultures to fluid shear in medium containing FITC-conjugated dextran (10 kDa). The proportion of dextran-retaining cells, indicative of transient membrane disruption and subsequent repair, was higher in microgravity-exposed osteocytes than controls, suggesting increased susceptibility to membrane damage upon reloading following disuse. Intracellular calcium signaling was assessed under a high but physiological fluid shear stress (30 dynes/cm^2^). Scaffolds cultured under disuse conditions demonstrated a larger sub-population of osteocytes with high calcium signaling intensity (F/Fo > 10 fold) during fluid shear. The maximum fold change in calcium signaling intensity over baseline and the duration of the peak calcium wave were greater for osteocytes cultured under disuse as compared to static controls, however the bioreactor-cultured osteocytes showed, on average, fewer calcium waves than those cultured under control conditions. Subsequent experiments demonstrated that the sub-population of osteocytes with high calcium signaling intensity following exposure to disuse were those that had experienced a transient membrane disruption event during reloading. Together, these results suggest that simulated microgravity enhances osteocyte susceptibility to formation of transient membrane damage and alters intracellular calcium signaling responses upon reloading. This integrated approach establishes a novel platform for mechanistic studies of osteocyte biology and could inform therapeutic strategies targeting skeletal disorders related to altered mechanical loading.

## 1. Introduction

Adaptation of the skeleton to mechanical loading is a key factor in bone health, as exercise promotes hypertrophy but disuse (such as from spaceflight) leads to bone loss. Bone loss on long-duration spaceflight missions, such as those intended for Mars, is a known problem at present, with astronauts losing approximately 1% to 1.5% of their bone mineral density in weight bearing locations per month [1,2,3]. Estimates based on computer simulations utilizing bone mineral density data from previous spaceflights suggest that 100% of conjunction-class (1000–1200 day mission length) astronauts on future Mars missions will develop osteopenia and nearly 80% will develop osteoporosis, losing up to 37% of their bone density in fracture prone sites critical for locomotion like the femoral neck [4]. Current countermeasures, such as resistance exercise, cannot fully abrogate bone loss during spaceflight [5,6]. Therefore, a critical need exists for better strategies to maintain bone health and prevent skeletal fractures in astronauts during weightlessness from spaceflight and upon return to gravitational forces. This, accordingly, necessitates a better understanding of mechanotransduction responses of osteocytes during reloading after disuse.

Osteocytes respond dynamically to mechanical stimuli, translating fluid shear stress into biochemical signals critical for bone homeostasis through a variety of mechano-sensing mechanisms. We and others [7,8,9,10,11,12] have previously observed that some osteocytes experience the formation of transient plasma membrane disruptions (PMDs) during exposure to high levels of in vitro and in vivo physiological loading, and that these PMDs are capable of initiating a calcium signaling-mediated mechanotransduction cascade. Importantly, we consistently observe that a small but detectable percentage of long bone osteocytes (~2% of the total osteocyte population, as estimated from our recent studies with improved imaging modalities [11]), show evidence of PMD formation with routine cage activity (i.e., under normal gravitational loading and locomotion) in mice, suggesting that formation of osteocyte PMDs may factor into bone’s sensation of and response to normal gravitational loads. However, the impact of disuse conditions on the formation of PMDs upon re-exposure to mechanical loading has not yet been tested.

Although osteocyte PMD formation has not yet been studied in the context of disuse, several previous studies have characterized the responses of osteocyte-like cells to microgravity conditions. Foundational studies from several decades ago hypothesized that osteocyte mechanotransduction would be impaired by microgravity [13] due to the osteocyte cytoskeleton being critical for mechanotransduction [14] and cytoskeleton dynamics being dependent upon gravity [15]. One of the first studies to report the actual effects of microgravity from spaceflight on osteocytes found evidence of enhanced osteolytic activity by mature osteocytes in iliac crest biopsies collected from rhesus macaques flown for 2 weeks on the BION-11 satellite [16]. These findings suggestive of osteocytic perilacunar remodeling activity were verified in female C57BL/6J mice subjected to microgravity for 15 days on the STS-131 space shuttle [17], and are consistent with a preprint reporting that female C57BL/6N mice flown on the STS-118 space shuttle mission demonstrated increased size of newly formed lacunae in compressively loaded bone locations (posterior lateral tibia) as compared to ground controls (Coulombe et al. [18]). Ocy454 cells exposed to up to 6 days of microgravity on the SpaceX Dragon-6 vessel showed impaired differentiation as well as alterations in cellular metabolic pathways such as glycolysis [19], but to date, direct investigation of mechanotransduction processes in osteocytes subjected to microgravity from spaceflight has not yet been reported.

Despite the scarcity of osteocyte mechanotransduction data from actual spaceflight experiments, our understanding of osteocytic responses to disuse has been advanced through ground-based models of simulated microgravity such as rotating wall vessel bioreactors which promote transcriptional changes like increased expression of *Sost* and *Tnfsf11* (Rankl) [19] that mimic the behavior of osteocytes subjected to reductions in mechanical loading in vivo [20,21,22]. A recent report utilizing MLO-Y4 osteocyte-like cells and a custom rotary clinostat suggested that exposure to disuse conditions compromised osteocyte calcium signaling responses triggered by exposure to fluid shear stress simulating reloading following disuse [23]. These results, although compelling and supported by an in vivo disuse/ex vivo loading model, utilized monolayer cell cultures and required intermediate cell handling steps such as trypsinizing and replating cells prior to induction of fluid flow that could have influenced downstream results [23]. Moreover, to the best of our knowledge, these findings have yet to be replicated by another investigator. To address these gaps in knowledge, and to better understand the potential impact of disuse conditions on the formation of osteocyte PMDs upon re-exposure to mechanical loading, we combined 3D osteocyte culture, microgravity simulation, fluid shear simulating reloading after disuse, PMD quantification, and real-time calcium signaling analysis to elucidate the responses of osteocytes under different mechanical environments.

## 2. Materials and Methods

### 2.1. Cell Line, Scaffold Preparation and 3D Culture Conditions

Ocy454 cells (a gift from Dr. Paola Divieti-Pajevic), which are capable of differentiating into a *Sost*-expressing osteocyte-like phenotype under the control of a temperature sensitive T-antigen, were cultured in growth medium consisting of α-MEM medium (Gibco 12561, Fisher Scientific, Waltham, MA, USA) + 10% FBS (Corning 35-010-CV, Lot #35010167, Fisher Scientific, Waltham, MA, USA) + 1% antibiotic/antimycotic (Corning 30-004-CI, Fisher Scientific, Waltham, MA USA) at their proliferative temperature of 33 °C in a humidified cell culture incubator (5% CO_2_) [21]. Scaffolds (Alvetex AVP004-48, REPROCELL USA, Beltsville, MD, USA) composed of porous polystyrene (200 µm thickness, 36-40 µm pore size) were rendered hydrophilic in 70% ethanol and rinsed in sterile PBS. Once hydrated, scaffolds were coated with a chilled 0.3 mg/mL type 1 collagen solution (Gibco A10483-01, Fisher Scientific, Waltham, MA, USA) suspended in cell culture grade water (Invitrogen 10977-015, Fisher Scientific, Waltham, MA, USA) at room temperature for one hour. Upon reaching 70% confluency, Ocy454 cells were treated with trypsin to dissociate them from the plate, seeded at a density of 1 × 10^6^ cells per scaffold and incubated at 33 °C for 90 min to promote cell attachment followed by addition of growth medium as described above. Cell-seeded scaffolds were cultured in 6 well plates at 33 °C in a humidified cell culture incubator for 3 days to promote proliferation, after which they were moved to a 37 °C humidified cell culture incubator for 10 to 12 days to promote osteocytic differentiation. 3D culture of Ocy454 cells on scaffolds as described here promoted robust development of a dendritic phenotype and increased expression of both *Sost* and *Tnfsf11* (Rankl) as compared to 2D culture conditions [21].

### 2.2. Exposure to Microgravity Conditions Simulating Disuse

Biopsy punches (Integra Miltex #33-32 0.3 cm, Fisher Scientific #12-460-406, Waltham, MA, USA) were utilized to produce 3 mm diameter cell-seeded scaffold constructs which were subjected to further culture at 37 °C in either 10 cm dishes (“Control”) for 72 h or in a rotating wall vessel bioreactor (“Disuse”; Synthecon Rotary Cell Culture System, Synthecon Incorporated, Houston, TX, USA) to simulate microgravity conditions. The bioreactor was set to 18.6 revolutions per minute (RPM) for 24 h after which rotation speed was increased to 20.9 RPM for an additional 48 h, as previously described, to maintain solid body rotation kinetics [19,21]. Although these rotating wall vessel bioreactor culture condition are known to generate a minimal amount of fluid shear stress (0.5 to 2 dynes/cm^2^) [21,24,25], previous studies have shown that exposure of Ocy454-seeded scaffolds to comparable levels of laminar fluid shear (in the absence of disuse) promotes decreased, rather than increased, expression of disuse-associated genes like *Sost* [21], validating the appropriateness of this model for investigating downstream effects of unloading. Scaffolds were either harvested immediately for analysis of gene expression or staining, or subjected to additional experimentation as described below. For visualization of dendritic morphology, scaffolds were fixed in a methanol-free formaldehyde solution in PBS for 15 min, washed, permeabilized with Triton X-100, and stained with an Alexa Fluor 488-labeled Phalloidin conjugate (Invitrogen A12379, Fisher Scientific, Waltham, MA, USA) to highlight F-actin in the Ocy454 cells. Nuclei were stained with Hoechst dye (Hoechst 33342, trihydrochloride trihydrate, Invitrogen H1399, Fisher Scientific, Waltham, MA, USA; 5 mg/mL stock solution diluted 1:1000 in PBS), after which scaffolds were mounted in glass slides with water-based mounting medium and imaged in a Nikon AX (Nikon Corporation, Melville, NY, USA) with NSPARC confocal microscope (20× objective, Z-stack image).

### 2.3. RNA Isolation and Gene Expression Analysis

For analysis of mRNA expression, scaffolds (*n* = 10 per culture condition for each biological replicate experiment; *n* = 6 biological replicates total) were transferred immediately from the bioreactor or static control culture plates into TRIzol reagent (15-596-018, Fisher Scientific, Waltham, MA, USA). Samples were incubated for 60 min on ice, after which total RNA was isolated and reverse transcribed into cDNA as previously described [12]. Briefly, RNA was reverse transcribed using a commercially available reverse transcription kit (Invitrogen Superscript III), and cDNA was subjected to semi-quantitative real-time PCR amplification (37.5 ng cDNA per 15 μL reaction volume reaction, run in triplicate) using a Bio-Rad CFX Connect system and SYBR green reagent (A25780, Fisher Scientific, Waltham, MA, USA). Gene expression levels were quantified using the comparative threshold cycle (2^−ΔΔCt^) method [26]. Expression of 18S rRNA was used as the internal control (housekeeping gene) for normalization. Primer sequences were as follows: 18S rRNA_F: 5′-AGTCCCTGCCCTTTGTACACA-3′, 18S rRNA_R: 5′-GGCCTCACTAAACCATCCAATC-3′, Sost_F: 5′-ACTTGTGCACGCTGCCTTCT-3′, Sost_R: 5′-TGACCTCTGTGGCATCATTCC-3′, Tnfsf11_F: 5′-GCTGGGACCTGCAAATAAGT-3′, Tnfsf11_R: 5′-TTGCACAGAAAACATTACACCTG-3′, Dkk1_F: 5′-TGCCTCCGATCATCAGACTGT-3′, Dkk1_R: 5′-CTTGGACCAGAAGTGTCTTGCA-3′. One biological replicate sample for each culture condition was subsequently dropped from the dataset due to unstable housekeeping gene expression, resulting in a final sample size of *n* = 5 biological replicates for each culture condition.

### 2.4. Turbulent Fluid Shear Stress (TFSS) and Osteocyte PMD Formation

Ocy454-seeded scaffolds (*n* = 10 per culture condition for each biological replicate experiment; *n* = 3 biological replicates total) were gently removed from the bioreactor or static control culture plates and placed in a 6 well plate containing a sterile solution of PBS + calcium chloride solution (21115-100mL, MilliporeSigma, Burlington, MA, USA; 1.8 mM final concentration of Ca^2+^) + 10 kDa FITC-dextran (1 mg/mL; 25 mL total volume). To induce turbulent fluid shear stress (TFSS), 10 mL of the PBS solution was withdrawn from the well with a serological pipet aid and rapidly dispensed on top of each scaffold approximately 1 inch above the well as previously described [11]. This procedure was repeated for a total of 5 cycles of TFSS per scaffold. Static control wells were subjected to one round of gentle media displacement (using the slowest setting on the pipet aid, with liquid displacement occurring at the edge of the well away from the scaffold). Following TFSS exposure, scaffolds were gently transferred to a new well containing PBS and Hoechst stain to visualize the cell nucleus (Hoechst 33342, trihydrochloride trihydrate, Invitrogen H1399, Fisher Scientific, Waltham, MA, USA; 5 mg/mL stock solution diluted 1:1000 in PBS). Scaffolds were incubated in this solution for 10 min in a 37 °C humidified incubator, gently rinsed with PBS and mounted to a glass slide using a water-based mounting medium (VectaMount AQ aqueous mounting medium, H-5501-60, Vector Laboratories, Newark, CA, USA). Scaffolds were imaged on a Leica STELLARIS confocal microscope using a 20× objective, and one z-stack image (30 micron depth) was collected for each scaffold. The abundance of transient plasma membrane disruptions (PMDs) formed during TFSS was quantified as previously described [12]. Cytosolic retention of FITC-dextran was interpreted as evidence of a repaired membrane disruption event; wounded cells permit entry of the dextran molecule, and successful PMD repair seals the tracer inside the cell [8]. The percentage of wounded osteocytes normalized to total cell number was quantified in each experiment.

### 2.5. Laminar Fluid Flow Shear Stress (LFSS)

Ocy454-seeded scaffolds were gently removed from the bioreactor (*n* = 6) or static control culture plates (*n* = 6) and placed to 1.5 mL tubes containing a Cal-520 AM dye solution (AAT Bioquest, Inc., Catalog #21130, suspended in DMSO + Hanks and HEPES Buffer Solution (HHBS; HEPES + CellGro 20-023-CV) + 0.04% Poloxamer188 (Fisher #AAJ6608736) and diluted 1:2 in media for a final Cal-520 dye concentration of 10 µM). Scaffolds were shielded from light and incubated in the Cal-520 solution at 37 °C for 90 min followed by room temperature for 30 min. Nuclei were stained using Hoechst dye (5 mg/mL stock solution diluted 1:1000 in PBS, introduced during the 30 minute room temperature incubation), after which scaffolds were gently rinsed three times with cell culture medium and once with PBS. Scaffolds were individually transferred to a Glycotech parallel plate flow chamber [8] on top of a rubber gasket which created a flow path (Type B, 0.010 inch thickness, 0.25 cm flow path) and covered with a glass slide which was clamped to the flow chamber assembly. Scaffolds were subjected to a unidirectional laminar fluid flow that induced a bulk laminar flow shear stress (LFSS) of 30 dynes/cm^2^ using a syringe pump (Harvard Apparatus PHD Ultra I/W) for a period of 5 min (300 s); flow medium consisted of sterile PBS + 1.8 mM calcium as described above for TFSS. Although fluid flow parameters were chosen to induce a bulk shear of 30 dynes/cm^2^ across the scaffolds in the flow chambers, it is important to note that computational modeling studies suggest that the 3D, porous nature of the scaffold architecture likely altered the exact shear stresses experienced by the Ocy454 osteocytes [27,28], and therefore the cells themselves may have experienced shear stresses outside of this min generalized value.

### 2.6. Calcium Signaling

Immediately prior to the onset of flow (~10 s) and throughout flow exposure, time-lapse calcium imaging was continuously recorded (FITC fluorescence settings; *n* = 1 image every 1.73 s) on a Leica STELLARIS microscope with a 10× objective lens. Cells attached to the 3D scaffolds exhibited stochastic and asynchronous calcium activations during flow stimulation, necessitating a customized analytical approach tailored to the unique mechanical and spatial context of scaffold-based cell growth. Due to subtle scaffold movement caused by fluid flow (Supplementary Figure S1), the calcium signal prior to the onset of flow was not used as a baseline reference. Instead, a baseline was defined for each individual cell region of interest (ROI) as the stable plateau period immediately preceding the highest calcium wave, visually identified from the fluorescence vs. time graph for each cell ROI. This approach accounts for dynamic signal drift and local motion artifacts within the scaffold. A total of *n* = 30 randomly selected osteocyte ROI’s were quantified for each scaffold. Calcium signaling metrics within each cell ROI were quantified from the fluorescence vs. time curves including maximum fold increase in Ca^2+^ fluorescence intensity over baseline (i.e., fold change for the highest calcium peak during the 300 s observation window) and duration of the highest peak (i.e., duration that fluorescence was above baseline for the highest peak during the 300 s observation window), after excluding ROI that did not demonstrate at least a 2-fold change in Cal-520 fluorescence over baseline. From these cell ROI values (*n* = 29 to 30 per scaffold, *n* = 6 scaffolds per culture condition), we calculated averages for each scaffold resulting in *n* = 6 biological replicate datasets per culture condition for (1) scaffold average maximum fluorescence fold change over baseline (F/Fo) and (2) scaffold average duration (in seconds) of the highest calcium wave peak. We also calculated (3) the average number of calcium waves over the 300 s observation window for each scaffold, where a calcium wave was again defined as at least a 2-fold change over baseline fluorescence (F/Fo).

In a subsequent set of experiments, we tested the relationship between osteocyte PMD formation and calcium signaling induced by LFSS following exposure to disuse as compared to static control culture conditions. Experiments as described above were repeated, but FITC-conjugated fluorescent dextran (10 kDa, Invitrogen D1820, Fisher Scientific, Waltham, MA, USA) was added to the flow medium to permit detection of cells experiencing a PMD, and the calcium indicator dye was changed to Cal-590 AM dye solution (20511, AAT Bioquest, Inc., Pleasanton, CA, USA, 10 µM; prepared and diluted as described above for Cal-520) to permit calcium signaling visualization in a different fluorescence spectrum. During flow experiments, calcium imaging was continuously recorded (Cy3 fluorescence settings; *n* = 1 image every 1.73 s, 5 min total recording) on a Leica STELLARIS microscope with a 10× objective lens. After calcium signaling data acquisition, the flow medium was changed to PBS containing 1.8 mM Ca^2+^, and the same LFSS settings were used to flush the dextran-containing medium from the scaffold. Static images were then captured using fluorescence settings for FITC (fluorescent dextran-containing cells), Cy3 (calcium), and Hoechst (nucleus). Cytosolic retention of FITC-dextran was interpreted as evidence of a repaired membrane disruption event. Calcium signaling metrics within cell ROI were quantified from the initial 5 min of flow as described above for the Cal-520 experiments, and were compared between cell ROI with and without evidence of PMD formation (*n* = 8 each).

### 2.7. Statistical Analysis

JMP Pro (v.18.0.2, JMP Statistical Discovery LLC, Cary, NC, USA) was used for all statistical analyses. A *p*-value of *p* ≤ 0.05 was considered to be statistically significant, and *p*-values reaching this threshold are presented in bold font in data figures. Comparisons of two groups (i.e., Control vs. Disuse) were made via Student’s *t*-tests. Comparisons of groups across two variables (i.e., culture condition: Control vs. Disuse, and PMD status: PMD+ or no PMD) were conducted with 2-factor ANOVA with interaction followed by Fisher’s LSD post hoc testing where appropriate. Statistical analyses for qPCR data were performed on delta delta CT values. Box plots in figures show median, quartiles, and outlier fences (where available) for each data set, where outlier fences represent first quartile –1.5 × (interquartile range) and third quartile +1.5 * (interquartile range). Unless otherwise noted in figure captions, each biological replicate in box plots is indicated by a separate data point, indicative of sample size for each data set.

## 3. Results

### 3.1. Establishment of the Disuse Bioreactor Model

**Figure 1:**
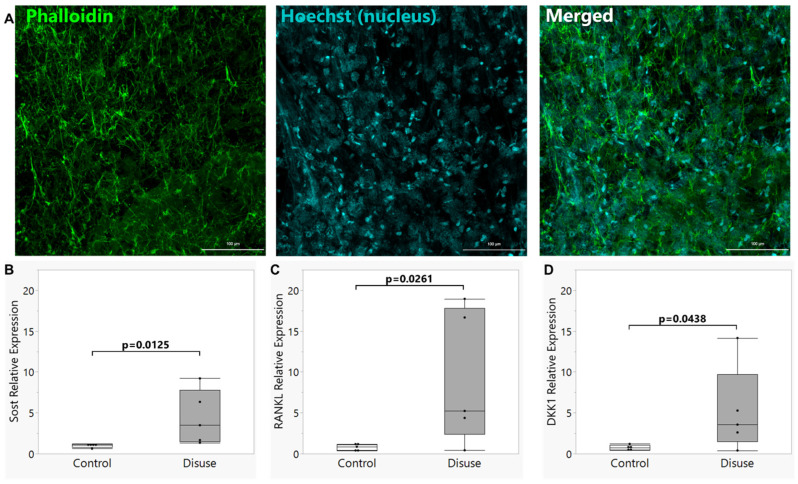
Visualization of the dendritic network and gene expression in scaffold-cultured Ocy454 osteocytes. (**A**) Phalloidin staining was performed to demonstrate the dendritic morphology of the 3D scaffold-cultured Ocy454 cells used in this study. (**B**–**D**) The mRNA expression levels of (**B**) *Sost*, (**C**) *Tnfsf11* (Rankl), and (**D**) *Dkk1* were all elevated in scaffolds seeded with Ocy454 osteocytes and cultured for 72 h under simulated microgravity conditions in a rotating wall vessel bioreactor (“Disuse”) as compared to similarly seeded scaffolds cultured under static control conditions (“Control”). Box plot shows median (middle line in box), quartiles (box bounds), and outlier fences for each group, where outlier fences represent first quartile −1.5 * (interquartile range) and third quartile +1.5 * (interquartile range), and each black circle represents one biological replicate experiment.

Comparable to previous reports [21], Ocy454 cells cultured under 3D conditions on the scaffolds developed a robust dendritic network (Figure 1A). Disuse promotes up-regulation of sclerostin [20,21] and Dkk1 [29,30], and it has been previously reported that exposure of Ocy454 osteocytes to microgravity-like conditions in a rotating wall vessel bioreactor up-regulates expression of genes like *Sost* and *Tnfsf11* (Rankl) [19,21,31]. To validate our use of this model, we seeded Ocy454 cells onto 3-dimensional scaffolds, differentiated them into a mature, osteocyte-like phenotype, and subjected them to 72 h of disuse in the bioreactor culture system. Expression level of both *Sost* and *Tnfsf11* (Rankl) were significantly higher in the bioreactor-cultured scaffolds as compared to static-cultured controls (*p* = 0.0125 and *p* = 0.0261, respectively; Figure 1B,C), as was expression of Dkk1 (*p* = 0.0438, Figure 1D), supporting the validity of our tissue culture model for inducing disuse-like responses in Ocy454 osteocytes.

### 3.2. Exposure to Disuse Sensitizes Osteocytes to the Formation of Membrane Damage upon Reloading

**Figure 2:**
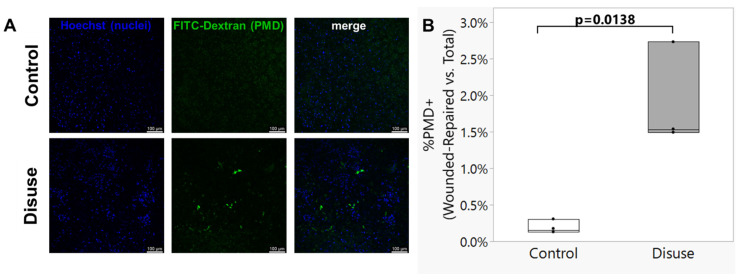
(**A**) Repaired plasma membrane disruptions (PMDs) formed by exposure to turbulent fluid shear stress were detected as cytosolic localization of 10 kDa FITC-dextran. (**B**) Ocy454-seeded scaffolds cultured for 72 h under simulated microgravity conditions (“Disuse”) demonstrated a greater abundance of repaired transient PMDs formed upon reloading via turbulent fluid shear stress as compared to static control cultures (“Control”) Box plot shows median (middle line), and each black circle represents one biological replicate experiment.

Although the 3D culture system utilized here, including exposure to microgravity-like conditions, was previously developed as reported by Spatz et al., it is important to note that cell culture constructs in the previous report were not subjected to subsequent mechanical loading. Therefore, the effects of reloading on the disuse-exposed 3D cultured osteocytes remained to be tested. Representative images from the turbulent fluid shear experiments are shown in Figure 2A. The number of osteocytes presenting with an intracellular signal for FITC-dextran, indicative of a transient plasma membrane disruption cell wounding event that was successfully repaired, following exposure to turbulent fluid flow shear stress was significantly greater for the scaffolds subjected to disuse as compared to control conditions (*p* = 0.0138; Figure 2B).

### 3.3. Exposure to Disuse Amplifies Calcium Signaling Responses upon Reloading

**Figure 3:**
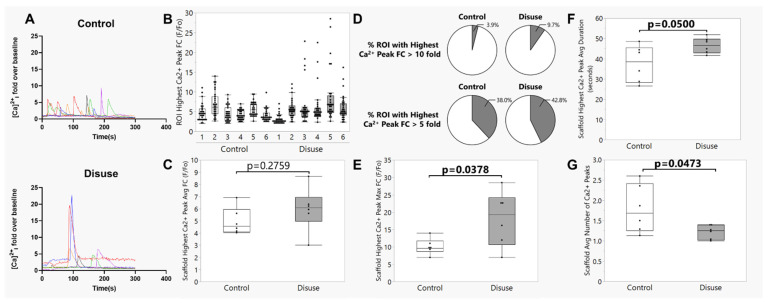
Calcium signaling dynamics of osteocytes subjected to microgravity-like conditions (disuse) are altered upon reloading. (**A**) One representative osteocyte cell ROI from each biological replicate scaffold was plotted as a fluorescence vs. time graph to illustrate calcium signaling dynamics associated with application of laminar fluid shear to Ocy454-seeded scaffolds subjected to microgravity (“Disuse”) or static control (“Control”) culture conditions prior to reloading. In each condition, independent replicate scaffolds are represented by different colors (red, orange, blue, green, purple, black). (**B**) Fold change (FC) in Cal-520 fluorescence, indicative of calcium signaling intensity, for the highest calcium wave visualized in each cell ROI during 5 min of laminar fluid shear; this was calculated as peak fluorescence (F) normalized to baseline fluorescence (Fo), where the latter was defined and quantified for each cell ROI immediately prior to the highest calcium wave. Each bar in the box plot represents one biological replicate scaffold (*n* = 6 total per culture condition), and each black dot represents one cell ROI. (**C**) Average fold change in Cal-520 fluorescence for the highest calcium wave experienced during 5 min of laminar fluid shear across each scaffold, calculated by averaging all ROIs measured for each scaffold. Note the existence of a small population of cells in the “Disuse” scaffolds with FC values > 10 that is largely absent from the “Control” group. (**D**) The percentage of cell ROI in each scaffold demonstrating a fold change (FC) in calcium signaling intensity greater than 10-fold (top panel) or 5-fold (bottom panel) for the highest Ca^2+^ peak. (**E**) Maximum fold change in Cal-520 fluorescence across each scaffold during 5 min of laminar fluid shear. (**F**) Average duration of the highest calcium wave experienced during 5 min of laminar fluid shear across each scaffold. (**G**) The average number of calcium waves observed over the 5 min of laminar fluid shear for each scaffold, where a calcium wave was defined as at least a 2-fold change in Cal-520 fluorescence over baseline fluorescence. Box plots show median (middle line), quartiles (box founds), and outlier fences for each scaffold (**B**) or group (**C**,**E**–**G**), where outlier fences represent first quartile −1.5 * (interquartile range) and third quartile +1.5 * (interquartile range).

Representative videos demonstrating the calcium signaling responses to induction of fluid shear stress can be seen in Supplementary Video S1 (static control culture conditions) and Supplementary Video S2 (disuse culture conditions). To illustrate calcium dynamics, one representative osteocyte cell ROI from each biological replicate scaffold was selected and plotted as a fluorescence vs. time graph (Figure 3A). The average peak fold change in Ca^2+^ fluorescence over baseline caused by laminar fluid shear stress was not different between static control and bioreactor-cultured scaffolds (Figure 3B,C, *p* = 0.2759). However, comparing across all osteocyte cell ROIs from each biological replicate scaffold, we noted that nearly every scaffold cultured under disuse conditions in the bioreactor demonstrated a sub-population of cells that showed a strikingly high calcium signaling intensity (F/Fo > 10 fold; Figure 3B,D); the size of this population was more than twice that found in scaffolds cultured under static control conditions (Figure 3D), even though few differences were noted between groups with a less stringent fold change threshold (F/Fo > 5 fold; Figure 3D). Consistent with this observation, the maximum (rather than the average) fold change in calcium signaling intensity over baseline across each scaffold was significantly greater for the constructs cultured under disuse conditions as compared to static controls (*p* = 0.0378, Figure 3E), as was the average duration of the highest calcium wave (*p* = 0.0500, Figure 3F). Although we unfortunately did not conduct live-dead staining of the osteocytes at the conclusion of flow, we examined the calcium signaling datasets for evidence of any osteocyte cell ROI demonstrating a persistent Cal-520 fluorescence signal that did not return to baseline, which would be suggestive of a persistent loss of cell membrane integrity (i.e., cell death). These analyses revealed very few osteocyte ROIs demonstrating this pattern of fluorescence (<10 total across the 360 cell ROIs randomly selected for analysis), with no differences in prevalence between static control or bioreactor-cultured scaffolds. Importantly, none of the few ROI with sustained Cal-520 fluorescence corresponded with the cell ROI exhibiting the pattern of high calcium signaling intensity in response to fluid shear (Figure 3E,F), suggesting that these higher-intensity calcium signaling dynamics were not associated with loss of cell viability. Interestingly, however, when considering the entire 300 s flow period, the bioreactor-cultured scaffolds showed, on average, a reduction in the total number of calcium waves experienced by osteocytes as compared to those cultured under static control conditions (*p* = 0.0473; Figure 3G).

### 3.4. The Amplified Calcium Signaling Responses in Disuse-Exposed Osteocytes Uniquely Occurred in Cells with Evidence of Membrane Damage

**Figure 4:**
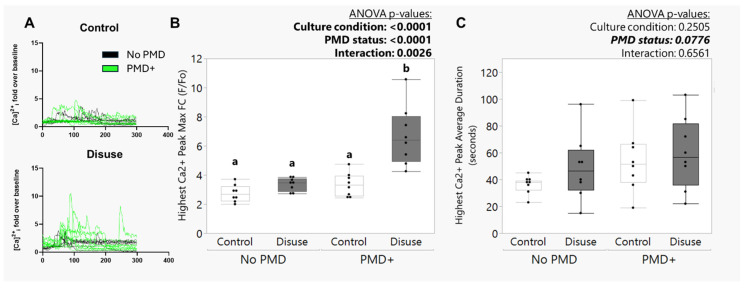
The amplified calcium signaling responses in disuse-exposed osteocytes uniquely occurred in cells with evidence of membrane damage. Laminar fluid shear stress calcium signaling experiments were conducted in the presence of a plasma membrane disruption (PMD) event tracer (10 kDa FITC-dextran). Calcium signaling dynamics were compared between osteocyte ROIs that were identified as experiencing a membrane disruption event (intracellular retention of FITC-dextran after exposure to flow; “PMD+”) and an equal number of randomly selected ROIs with no evidence of plasma membrane damage (“No PMD”). (**A**) Osteocyte cell ROI were plotted as a fluorescence vs. time graph to illustrate calcium signaling dynamics associated with application of laminar fluid shear to Ocy454-seeded scaffolds subjected to microgravity (“Disuse”) or static control (“Control”) culture conditions prior to reloading. (**B**) Maximum fold change in Cal-520 fluorescence during 5 min of laminar fluid shear across the quantified ROI. A synergistic interaction between microgravity conditions and membrane wounding during reloading that impacted the downstream magnitude of calcium signaling was detected. Letters above each bar denote results of Fisher’s LSD posthoc testing; bars with different superscript letters are significantly (*p* < 0.05) different from one another. (**C**) The average duration of the highest calcium wave experienced during 5 min of laminar fluid shear across the quantified ROI was not significantly different between groups. Each black dot represents one cell ROI.

To test whether the association between exposure to disuse and high calcium signaling intensity during reloading was related to membrane wounding, laminar fluid shear stress calcium signaling experiments were repeated in the presence of the membrane disruption event tracer (10 kDa FITC-dextran). Calcium signaling dynamics were compared between osteocyte ROIs that were identified as experiencing a membrane disruption event (intracellular retention of FITC-dextran after exposure to flow) and an equal number of randomly selected ROIs with no evidence of plasma membrane damage. To illustrate calcium dynamics, osteocyte cell ROIs with evidence of plasma membrane disruption (PMD+) and no evidence of plasma membrane disruption (No PMD) were plotted as a fluorescence vs. time graph (Figure 4A). We observed a statistically significant interaction effect between exposure to microgravity conditions and PMD formation, where the maximum fold change in calcium signaling intensity over baseline was only significantly greater for the disuse-exposed osteocytes with evidence of membrane wounding during reloading (*p*_interaction_ = 0.0026, Figure 4B). This suggests the presence of a synergistic interaction between microgravity conditions and membrane wounding during reloading that impacts the downstream magnitude of calcium signaling. Interestingly, although we observed a microgravity-related increase in the average duration of the highest calcium wave in earlier experiments (Figure 3F), this trend did not reach statistical significance in the follow-up experiments with the FITC-dextran PMD tracer where a smaller number of ROIs were considered (Figure 4C). However, we did note a mild trend for PMD formation to be associated with a longer calcium wave duration (*p* = 0.0776; Figure 4C) as compared to non-wounded cells. The total number of calcium waves experienced by osteocytes during reloading was not affected by PMD formation (*p* = 0.7286) nor exposure to microgravity conditions (*p* = 0.3021) in these experiments.

## 4. Discussion

The primary impact of the current study is the successful development of an in vitro culture system permitting direct and rapid analysis of the impact of adaptation to disuse conditions on osteocytic mechanotransduction responses during subsequent reloading. While similar in vitro models have already been established for investigating effects of disuse on osteocytes [19,21], a novel contribution of the current study is the ability to subject disuse-exposed osteocytes to immediate reloading via fluid shear. Previous studies have demonstrated the utility of calcium indicator mice (e.g., GCaMP3, GCaMP6 models) to permit real-time visualization of load-induced calcium signaling dynamics in situ in living animals [32,33]. These models demonstrate that loading-induced calcium signals in osteocytes encode strain magnitude, whereby the number of osteocytes initiating calcium signaling (but not the intensity of the calcium signal itself in discrete cells) correlates strongly to loading frequency [34], but to the best of our knowledge, to date these models have not been subjected to disuse-like conditions in vivo. Our model suggests that microgravity-like conditions induce a heterogeneous response that renders a population of osteocytes more susceptible to the formation of plasma membrane damage upon reloading. Moreover, our data also suggest that these disuse-adapted osteocytes experiencing membrane damage during reloading demonstrate amplified calcium signaling responses as compared to non-wounded cells. Therefore, although PMD formation is only one of many potential upstream mechanosensation events that trigger downstream calcium signaling in osteocytes [35,36,37], this mechanism appears particularly responsive to the impact of altered gravitational loading on osteocyte mechanotransduction.

Somewhat comparable to our approach here, an intriguing recent study also investigated the impact of disuse and subsequent reloading on osteocytic calcium responses. Liu et al. subjected T-25 flasks of MLO-Y4 or primary osteocytes to 48 h of disuse from simulated microgravity in a custom clinostat bioreactor [38], after which cells were trypsinized, replated on to collagen-coated glass slides for 24 h, and subjected to 2 Pa (i.e., 20 dynes/cm^2^) laminar fluid shear for 10 min in a parallel plate flow chamber using a peristaltic pump [23]. Carrying these studies in vivo, they also subjected 3-month-old male C57BL/6J mice to 4 weeks of hindlimb unloading. After the disuse period, mice were sacrificed, tibias were isolated and incubated in cell culture media for 3 h (including a 1 h period for loading with Cal-520 calcium indicator dye), and subjected to ex vivo tibial loading [39] while imaging osteocyte calcium signaling dynamics in situ. These studies, like ours, suggest that exposure to disuse conditions promotes an overall reduction in the number of calcium waves experienced by osteocytes subjected to reloading (Figure 3G). However, Liu et al. found that the intensity of the flow-induced Ca^2+^ wave was blunted in osteocytes (but not osteoblasts) subjected to microgravity as compared to control conditions [23]. These changes were described as related to disuse-induced alterations in a HIF-1α/pyruvate dehydrogenase kinase 1 (PDK1) axis-mediated increased dependency on glutamine metabolism, glutamine exhaustion, and glycolytic metabolic behavior driving reduced ATP synthesis. In contrast, our results suggest that while average calcium wave intensity was unchanged, scaffolds cultured under disuse conditions in the bioreactor contained a unique sub-population of osteocytes which demonstrated an amplified, rather than a blunted, response to fluid shear (Figure 3D–F and Figure 4). Both the relative number of osteocytes experiencing a transient PMD in response to fluid shear (Figure 2) and the relative number of osteocytes demonstrating an exceptionally high Ca^2+^ signaling intensity in response to fluid shear (Figure 3D,E) were increased for the scaffolds subjected to disuse culture conditions. Critically, subsequent studies (Figure 4) support the conclusion that these osteocytes which developed PMDs with fluid shear following disuse are those that show the amplified calcium signaling response.

At present, we cannot fully explain the discrepancy between our results and those of Liu et al. [23], although we wish to highlight important differences in our experimental conditions that may have contributed to dissimilarities in outcome measures. In particular, our model utilizing culture on 3D scaffolds facilitated direct and rapid movement of scaffolds from disuse to reloading conditions without requiring cell trypsinization or replating, whereas those from Liu et al. were trypsinized, re-seeded onto glass slides, and cultured for 24 h prior to flow exposure. Both older [40,41] and more recent [42] studies show that trypsinization of anchorage-dependent cell types promotes profound changes in the cell cytoskeletal arrangement that leads to cell rounding and detachment, and detachment of cells from their substrate alters cell stiffness [43]. This is critical because disuse conditions also promote cytoskeletal rearrangement in mechanically sensitive cell populations like endothelial cells, which like osteocytes can experience transient plasma membrane damage in a dose-responsive fashion to shear [44]. This cytoskeletal rearrangement in endothelial cells promotes cell rounding and decreased cell stiffness, with particularly rapid changes taking place at the cell cortex [45]. Osteoblast-like cells, too, have been reported to experience cell rounding in response to disuse [46,47,48]. These same cells also showed larger protrusion lengths into microfluidic pipettes during microgravity suggestive of decreased stiffness, and lower filamentous actin (F-actin) with greater actin disassembly as compared to ground controls [49]. Gene sets from Ocy454 cells subjected to 6 days of microgravity conditions aboard the SpaceX Dragon Commercial Resupply Service Mission 6 (CRS-6) also suggested altered cellular function associated with cytoskeleton dynamics (e.g., “organization of cytoskeleton” and “microtubule dynamics”; [19]). Thus, the intermediate step of trypsinizing and replating cells prior to reloading studies in the Liu et al. study [23], combined with other experimental differences such as cell line (MLO-Y4 vs. Ocy454), magnitude of shear stress (20 dynes/cm^2^ vs. 30 dynes/cm^2^), and model of microgravity (clinostat vs. rotating wall vessel bioreactor) may have influenced the downstream results and contributed to the discrepancy between our studies. Our inability to establish a conclusive explanation for the disagreement between our findings and those of Liu et al. remains a limitation of our study.

Older literature reports that exposure to disuse conditions can sensitize skeletal muscle cells to mechanically induced membrane damage upon reloading. This has been shown for male humans subjected to bed rest [50], adult female Wistar rats subjected to hindlimb unloading [51], male Sprague Dawley rats subjected to hindlimb suspension [52], and male Sprague Dawley rats subjected to spaceflight [53]. These results are consistent with the findings of the current study, where osteocyte-laden scaffolds subjected to 72 h of disuse formed more transient PMDs upon reloading as compared to scaffolds cultured under static control conditions. In a recent study, 14 days of disuse via tail suspension tended to reduce cortical bone mass (cortical bone area, thickness, section modulus) and mechanical properties (stiffness, modulus) in 18 month old male mice, and this cortical bone loss was surprisingly amplified rather than recovered following 2- or 14-days of reloading via normal cage activity [54]. Moreover, although disuse did not promote immediate loss of osteocyte viability (as visualized by TUNEL staining) in these 18 month old mice, decreased osteocyte viability was seen at the conclusion of 14 days of reloading following disuse [54]. These findings may be consistent with the idea that exposure to reduced mechanical loading is capable of predisposing osteocytes to cell death upon reloading. However, we observed very few osteocyte ROIs with sustained Cal-520 fluorescence suggestive of osteocyte cell death, and no apparent differences in the relative abundance of these sparsely observed cells between disuse and control culture conditions. Moreover, although evidence of cell membrane damage (PMD) from reloading was increased by disuse culture conditions, detection of these events necessitates successful PMD repair to seal the tracer inside the cell which supports a conclusion of post-wounding cell survival rather than cell death [11,12]. Therefore, our studies at present do not support a connection between cell membrane damage formed during reloading and subsequent loss of osteocyte viability, suggesting that the decreased osteocyte viability previously reported during reloading may be attributable to other mechanisms. However we note that our loading studies were conducted over a very short period of time (~5 min of fluid flow) after a comparatively short exposure to disuse conditions (72 h). Longer-term studies that include post-flow incubation of disuse and control cultures for various lengths of time, coupled with live-dead staining, would be an important next step for determining whether exposure to microgravity renders osteocytes more likely to sustain deleterious damage upon reloading.

## 5. Conclusions

In conclusion, studies shown here demonstrate the utility of a novel model that integrates 3D culture and live calcium imaging during loading to understand osteocyte mechanotransduction pathways. Together, these results suggest that simulated microgravity enhances osteocyte membrane susceptibility to the formation of transient damage and alters intracellular calcium signaling responses upon reloading. This integrated approach establishes a platform for mechanistic studies of osteocyte biology and could inform therapeutic strategies targeting skeletal disorders related to altered mechanical loading. While our results support an improved understanding of mechanotransduction responses of osteocytes during reloading after disuse, it will be important to test these paradigms in more translationally relevant models (e.g., use of primary osteocytes, human cells lines, and/or in vivo models) before extrapolating these findings to important topics such as consideration of astronaut health.

## Data Availability

The data that support the findings of this study are available from the corresponding author upon reasonable request.

## Supplementary Materials

The following supporting information can be downloaded at: https://www.mdpi.com/article/10.3390/biom15111534/s1, Figure S1: Representative image of Cal-520 fluorescence, indicative of calcium signaling intensity, from a scaffold during exposure to laminar fluid shear; Video S1: Static control culture conditions; Video S2: disuse culture conditions.

## References

[1] Lang T., LeBlanc A., Evans H., Lu Y., Genant H., Yu A. (2004). Cortical and trabecular bone mineral loss from the spine and hip in long-duration spaceflight. J. Bone Miner. Res. Off. J. Am. Soc. Bone Miner. Res..

[2] LeBlanc A., Schneider V., Shackelford L., West S., Oganov V., Bakulin A., Voronin L. (2000). Bone mineral and lean tissue loss after long duration space flight. J. Musculoskelet. Neuronal Interact..

[3] LeBlanc A.D., Spector E.R., Evans H.J., Sibonga J.D. (2007). Skeletal responses to space flight and the bed rest analog: A review. J. Musculoskelet. Neuronal Interact..

[4] Axpe E., Chan D., Abegaz M.F., Schreurs A.S., Alwood J.S., Globus R.K., Appel E.A. (2020). A human mission to Mars: Predicting the bone mineral density loss of astronauts. PLoS ONE.

[5] Sibonga J., Matsumoto T., Jones J., Shapiro J., Lang T., Shackelford L., Smith S.M., Young M., Keyak J., Kohri K. (2019). Resistive exercise in astronauts on prolonged spaceflights provides partial protection against spaceflight-induced bone loss. Bone.

[6] Laurens C., Simon C., Vernikos J., Gauquelin-Koch G., Blanc S., Bergouignan A. (2019). Revisiting the Role of Exercise Countermeasure on the Regulation of Energy Balance During Space Flight. Front. Physiol..

[7] Hagan M.L., Bahraini A., Pierce J.L., Bass S.M., Yu K., Elsayed R., Elsalanty M., Johnson M.H., McNeil A., McNeil P.L. (2018). Inhibition of Osteocyte Membrane Repair Activity via Dietary Vitamin E Deprivation Impairs Osteocyte Survival. Calcif. Tissue Int..

[8] Yu K., Sellman D.P., Bahraini A., Hagan M.L., Elsherbini A., Vanpelt K.T., Marshall P.L., Hamrick M.W., McNeil A., McNeil P.L. (2018). Mechanical loading disrupts osteocyte plasma membranes which initiates mechanosensation events in bone. J. Orthop. Res..

[9] Mikolajewicz N., Zimmermann E.A., Willie B.M., Komarova S.V. (2018). Mechanically-stimulated ATP release from murine bone cells is regulated by a balance of injury and repair. eLife.

[10] Hagan M.L., Yu K., Zhu J., Vinson B.N., Roberts R.L., Montesinos Cartagena M., Johnson M.H., Wang L., Isales C.M., Hamrick M.W. (2020). Decreased pericellular matrix production and selection for enhanced cell membrane repair may impair osteocyte responses to mechanical loading in the aging skeleton. Aging Cell.

[11] Tuladhar A., Shaver J.C., McGee W.A., Yu K., Dorn J., Horne J.L., Alhamad D.W., Hagan M.L., Cooley M.A., Zhong R. (2024). Prkd1 regulates the formation and repair of plasma membrane disruptions (PMD) in osteocytes. Bone.

[12] Hagan M.L., Tuladhar A., Yu K., Alhamad D.W., Bensreti H., Dorn J., Piedra V.M., Cantu N., Stokes E.G., Blumenthal D. (2024). Osteocyte Sptbn1 Deficiency Alters Cell Survival and Mechanotransduction Following Formation of Plasma Membrane Disruptions (PMD) from Mechanical Loading. Calcif. Tissue Int..

[13] Burger E.H., Klein-Nulend J. (1998). Microgravity and bone cell mechanosensitivity. Bone.

[14] Ajubi N.E., Klein-Nulend J., Nijweide P.J., Vrijheid-Lammers T., Alblas M.J., Burger E.H. (1996). Pulsating fluid flow increases prostaglandin production by cultured chicken osteocytes--a cytoskeleton-dependent process. Biochem. Biophys. Res. Commun..

[15] Tabony J., Job D. (1992). Gravitational symmetry breaking in microtubular dissipative structures. Proc. Natl. Acad. Sci. USA.

[16] Rodionova N.V., Oganov V.S., Polkovenko O.V. (2002). Mechanisms of gravity-dependent changes in the bone tissue. J. Gravit. Physiol..

[17] Blaber E.A., Dvorochkin N., Lee C., Alwood J.S., Yousuf R., Pianetta P., Globus R.K., Burns B.P., Almeida E.A. (2013). Microgravity induces pelvic bone loss through osteoclastic activity, osteocytic osteolysis, and osteoblastic cell cycle inhibition by CDKN1a/p21. PLoS ONE.

[18] Coulombe J.C., Mullen Z.A., Weins A.M., Fisher L.E., Lynch M.E., Stodieck L.S., Ferguson V.L. (2022). Reduced local mechanical stimuli in spaceflight diminishes osteocyte lacunar morphometry and spatial heterogeneity in mouse cortical bone. bioRxiv.

[19] Uda Y., Spatz J.M., Hussein A., Garcia J.H., Lai F., Dedic C., Fulzele K., Dougherty S., Eberle M., Adamson C. (2021). Global transcriptomic analysis of a murine osteocytic cell line subjected to spaceflight. FASEB J..

[20] Spatz J.M., Fields E.E., Yu E.W., Divieti Pajevic P., Bouxsein M.L., Sibonga J.D., Zwart S.R., Smith S.M. (2012). Serum sclerostin increases in healthy adult men during bed rest. J. Clin. Endocrinol. Metab..

[21] Spatz J.M., Wein M.N., Gooi J.H., Qu Y., Garr J.L., Liu S., Barry K.J., Uda Y., Lai F., Dedic C. (2015). The Wnt Inhibitor Sclerostin Is Up-regulated by Mechanical Unloading in Osteocytes in Vitro. J. Biol. Chem..

[22] Cabahug-Zuckerman P., Frikha-Benayed D., Majeska R.J., Tuthill A., Yakar S., Judex S., Schaffler M.B. (2016). Osteocyte Apoptosis Caused by Hindlimb Unloading is Required to Trigger Osteocyte RANKL Production and Subsequent Resorption of Cortical and Trabecular Bone in Mice Femurs. J. Bone Miner. Res. Off. J. Am. Soc. Bone Miner. Res..

[23] Liu X., Yan Z., Cai J., Wang D., Yang Y., Ding Y., Shao X., Hao X., Luo E., Guo X.E. (2023). Glucose- and glutamine-dependent bioenergetics sensitize bone mechanoresponse after unloading by modulating osteocyte calcium dynamics. J. Clin. Investig..

[24] Hammond T.G., Hammond J.M. (2001). Optimized suspension culture: The rotating-wall vessel. Am. J. Physiol. Ren. Physiol..

[25] Song K., Wang H., Zhang B., Lim M., Liu Y., Liu T. (2013). Numerical simulation of fluid field and in vitro three-dimensional fabrication of tissue-engineered bones in a rotating bioreactor and in vivo implantation for repairing segmental bone defects. Cell Stress Chaperones.

[26] McGee-Lawrence M.E., Carpio L.R., Schulze R.J., Pierce J.L., McNiven M.A., Farr J.N., Khosla S., Oursler M.J., Westendorf J.J. (2016). Hdac3 Deficiency Increases Marrow Adiposity and Induces Lipid Storage and Glucocorticoid Metabolism in Osteochondroprogenitor Cells. J. Bone Miner. Res..

[27] Verbruggen S.W., Vaughan T.J., McNamara L.M. (2014). Fluid flow in the osteocyte mechanical environment: A fluid-structure interaction approach. Biomech. Model. Mechanobiol..

[28] Price C., Zhou X., Li W., Wang L. (2011). Real-time measurement of solute transport within the lacunar-canalicular system of mechanically loaded bone: Direct evidence for load-induced fluid flow. J. Bone Miner. Res. Off. J. Am. Soc. Bone Miner. Res..

[29] Frings-Meuthen P., Boehme G., Liphardt A.M., Baecker N., Heer M., Rittweger J. (2013). Sclerostin and DKK1 levels during 14 and 21 days of bed rest in healthy young men. J. Musculoskelet. Neuronal Interact..

[30] Belavy D.L., Baecker N., Armbrecht G., Beller G., Buehlmeier J., Frings-Meuthen P., Rittweger J., Roth H.J., Heer M., Felsenberg D. (2016). Serum sclerostin and DKK1 in relation to exercise against bone loss in experimental bed rest. J. Bone Miner. Metab..

[31] Nakagaki R., Mukaibo T., Monir A., Gao X., Munemasa T., Nodai T., Tamura A., Obikane Y.H., Kondo Y., Masaki C. (2024). Simulated microgravity environment inhibits matrix mineralization during the osteoblast to osteocyte differentiation. Biochem. Biophys. Res. Commun..

[32] Lewis K.J., Boorman-Padgett J.F., Castaneda M., Spray D.C., Thi M.M., Schaffler M.B. (2023). A Fluorescent Intravital Imaging Approach to Study Load-Induced Calcium Signaling Dynamics in Mouse Osteocytes. J. Vis. Exp..

[33] Lewis K.J., Cabahug-Zuckerman P., Boorman-Padgett J.F., Basta-Pljakic J., Louie J., Stephen S., Spray D.C., Thi M.M., Seref-Ferlengez Z., Majeska R.J. (2021). Estrogen depletion on In vivo osteocyte calcium signaling responses to mechanical loading. Bone.

[34] Lewis K.J., Frikha-Benayed D., Louie J., Stephen S., Spray D.C., Thi M.M., Seref-Ferlengez Z., Majeska R.J., Weinbaum S., Schaffler M.B. (2017). Osteocyte calcium signals encode strain magnitude and loading frequency in vivo. Proc. Natl. Acad. Sci. USA.

[35] Qin L., Liu W., Cao H., Xiao G. (2020). Molecular mechanosensors in osteocytes. Bone Res..

[36] Li X., Kordsmeier J., Xiong J. (2021). New Advances in Osteocyte Mechanotransduction. Curr. Osteoporos. Rep..

[37] Hagan M.L., Balayan V., McGee-Lawrence M.E. (2021). Plasma membrane disruption (PMD) formation and repair in mechanosensitive tissues. Bone.

[38] Hu Z., Wang Y., Sun Z., Wang H., Zhou H., Zhang L., Zhang S., Cao X. (2015). miRNA-132-3p inhibits osteoblast differentiation by targeting Ep300 in simulated microgravity. Sci. Rep..

[39] Morrell A.E., Robinson S.T., Silva M.J., Guo X.E. (2020). Mechanosensitive Ca^2+^ signaling and coordination is diminished in osteocytes of aged mice during ex vivo tibial loading. Connect. Tissue Res..

[40] Badley R.A., Woods A., Carruthers L., Rees D.A. (1980). Cytoskeleton changes in fibroblast adhesion and detachment. J. Cell Sci..

[41] Raizada M.K., Tan G., Fellows R.E. (1981). Trypsin-induced alterations of insulin binding, microfilament organization and cell shape in fibroblastic cultures from non-diabetic and diabetic mice. Exp. Cell Res..

[42] Lordon B., Campion T., Gibot L., Gallot G. (2024). Impact of trypsin on cell cytoplasm during detachment of cells studied by terahertz sensing. Biophys. J..

[43] Nehls S., Noding H., Karsch S., Ries F., Janshoff A. (2019). Stiffness of MDCK II Cells Depends on Confluency and Cell Size. Biophys. J..

[44] Raj N., Pan C., Starke A.M., Matos A.L.L., Soehnlein O., Gerke V. (2025). Altered shear stress of blood flow causes plasma membrane damage in endothelial cells. Blood Vessel. Thromb. Hemost..

[45] Janmaleki M., Pachenari M., Seyedpour S.M., Shahghadami R., Sanati-Nezhad A. (2016). Impact of Simulated Microgravity on Cytoskeleton and Viscoelastic Properties of Endothelial Cell. Sci. Rep..

[46] Wu X.T., Yang X., Tian R., Li Y.H., Wang C.Y., Fan Y.B., Sun L.W. (2022). Cells respond to space microgravity through cytoskeleton reorganization. FASEB J. Off. Publ. Fed. Am. Soc. Exp. Biol..

[47] Hughes-Fulford M. (2003). Function of the cytoskeleton in gravisensing during spaceflight. Adv. Space Res..

[48] Guignandon A., Vico L., Alexandre C., Lafage-Proust M.H. (1995). Shape changes of osteoblastic cells under gravitational variations during parabolic flight—Relationship with PGE2 synthesis. Cell Struct. Funct..

[49] Wubshet N.H., Cai G., Chen S.J., Sullivan M., Reeves M., Mays D., Harrison M., Varnado P., Yang B., Arreguin-Martinez E. (2024). Cellular mechanotransduction of human osteoblasts in microgravity. NPJ Microgravity.

[50] Clarke M.S., Bamman M.M., Feeback D.L. (1998). Bed rest decreases mechanically induced myofiber wounding and consequent wound-mediated FGF release. J. Appl. Physiol. (1985).

[51] Kasper C.E. (1995). Sarcolemmal disruption in reloaded atrophic skeletal muscle. J. Appl. Physiol. (1985).

[52] Krippendorf B.B., Riley D.A. (1993). Distinguishing unloading- versus reloading-induced changes in rat soleus muscle. Muscle Nerve.

[53] Riley D.A., Ellis S., Slocum G.R., Sedlak F.R., Bain J.L., Krippendorf B.B., Lehman C.T., Macias M.Y., Thompson J.L., Vijayan K. (1996). In-flight and postflight changes in skeletal muscles of SLS-1 and SLS-2 spaceflown rats. J. Appl. Physiol. (1985).

[54] Cunningham H.C., Orr S., Murugesh D.K., Hsia A.W., Osipov B., Go L., Wu P.H., Wong A., Loots G.G., Kazakia G.J. (2023). Differential bone adaptation to mechanical unloading and reloading in young, old, and osteocyte deficient mice. Bone.

