# Supplementary material for: Integrating 3D Osteocyte Culture, Microgravity Simulation, and Fluid Flow Reveals Mechanisms of Osteocyte Mechanosensation and Calcium Signaling Altered by Disuse
Source: Biomolecules. 2025 Oct 31;15(11):1534. doi: 10.3390/biom15111534 (PMC12649886; doi:10.3390/biom15111534)
Supplement: Supplementary file 1 [file biomolecules-15-01534-s001.zip › Supplementary Materials.pdf]

Supplementary Materials

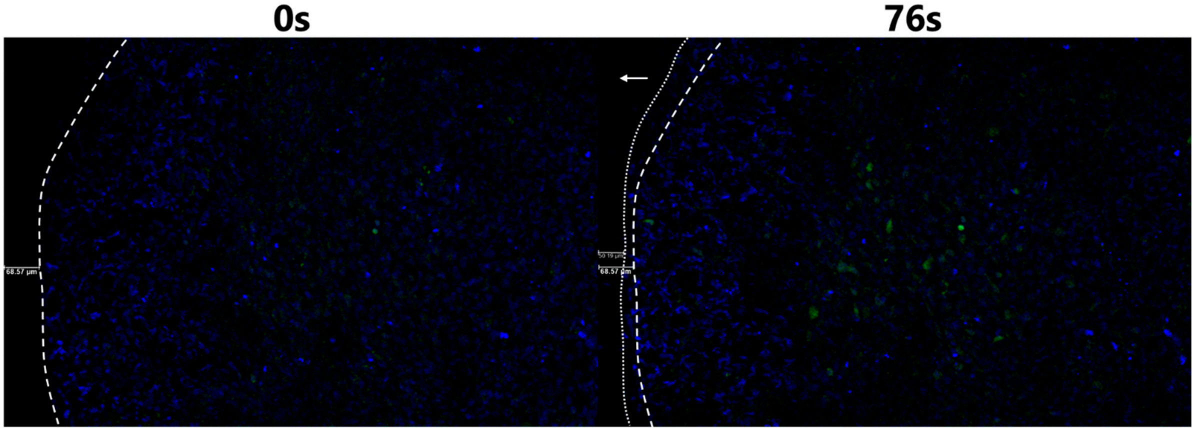

Figure S1. Representative image of Cal-520 fluorescence, indicative of calcium signaling intensity, from a scaffold during exposure to laminar fluid shear. Still images from the video captured at 0 seconds (i.e., onset of flow) and 76 seconds (i.e., after 76 seconds of exposure to 30 dynes/cm^2 shear stress) demonstrate subtle movement of the scaffold over time; scaffold border is indicated by the white hashed line in both images, and original location of the scaffold is indicated by the dotted white line in the image collected after 76 seconds of flow. Due to this subtle movement, a baseline fluorescence (Fo) value was established for each cell ROI immediately prior to the highest calcium wave experience during the 5 minutes of laminar fluid shear, rather than normalizing each cell ROI to the baseline fluorescence visible prior to the onset of flow. .

Supplementary Video S1: Control.

Calcium signaling dynamics in Ocy454 osteocytes cultured under static control conditions during exposure to laminar fluid shear stress (30 dynes/cm^2).

Ocy454 osteocytes seeded in 3D Alvetex scaffolds were loaded with Cal-520 AM calcium indicator dye and subjected to laminar fluid shear stress for 5 minutes. The experiment was recorded at 1.73 seconds per frame, and the playback video is shown at 4 frames per second. Green fluorescence indicates transient increases in intracellular calcium concentration (Cal-520), while blue fluorescence (Hoechst) marks nuclei.

Supplementary Video S2: Disuse.

Calcium signaling dynamics in Ocy454 osteocytes cultured under simulated microgravity (disuse) conditions during exposure to laminar fluid shear stress (30 dynes/cm^2).

Ocy454 osteocytes cultured for 72 hours under simulated microgravity in a rotating wall vessel bioreactor were subjected to laminar fluid shear stress while loaded with Cal-520 AM dye. The experiment was recorded at 1.73 seconds per frame, and the playback video is shown at 4 frames per second. Green fluorescence (Cal-520) represents calcium transients, while blue fluorescence (Hoechst) marks nuclei.
